# Gametogenic cycle of *Crassostrea gigas* in contrasting Mediterranean habitats: marine (Gulf of Tunis) and continental (Bizert lagoon) culture sites

**DOI:** 10.1186/2241-5793-21-13

**Published:** 2014-07-02

**Authors:** Salwa Dridi, Mohamed Salah Romdhane, M’hamed Elcafsi

**Affiliations:** Institut Supérieur de Pêche et d’Aquaculture de Bizerte, ISPA –BP 15, ERRIMEL, 7080 Bizerte, Tunisia; Département des Sciences de la, Production Animale et de la Pêche, Unité d’écosystèmes et ressources aquatiques, Institut National Agronomique de Tunisie, 43 Av. Charles Nicole, 1082 Tunis, Tunisia; Faculté des Sciences de Tunis/ Département de Biologie, Unité de Physiologie et d’Ecophysiologie des Organismes Aquatiques, Campus Universitaire, 2092, El Manar II, Tunis, Tunisia

**Keywords:** Oysters, Gametogenesis, Chlorophyll *a*, Farming

## Abstract

**Background:**

The gametogenic cycle of *Crassostrea gigas*, a species imported into the Mediterranean for aquaculture, has been studied (May 2005 to July 2006) in two contrasting habitats of Tunisia: the Bizert lagoon, where oyster farms have been developed since 1970, and the Gulf of Tunis, where oysters have been experimentally farmed during this study, to assess the potential of this latter marine area for sustaining oyster-culture.

**Results:**

The sexual cycle of the species was described through the histological examination of the gonads, the estimation of oocytes diameter, and the assessment of its condition and gonadal condition indices. The applied techniques gave similar results. The gametogenic cycle of *C. gigas* was precocious and more intense in oysters farmed within the lagoon than in the marine area, considering as well gonadal growth, maturation stages and gametes release.

**Conclusions:**

The obtained results are probably related with the different environmental conditions of the studied habitats, temperature and food supply, in particular. The sexual cycle of the species was successfully completed in the marine area, stressing the invasive character of *C. gigas*.

## Background

Oyster culture is a rather recent activity in Tunisia developed in the beginning of the previous century, where a breeding attempt of the species *Crassostrea angulata* in the lagoon of Ghar El Melh, in 1931, was successful accomplished [1]. The first farms were deployed in the 1950s in the Bizert lagoon by an Italian farmer who imported spats of the Portuguese oyster breeding from France [1]. Until 1972, only *C. angulata* was cultured in the area, but after the collapse of the species populations in Europe due to an outbreak, the supply of natural spats ceased. Thus, ONP (Office National de Pêche) tried the breeding of the Japanese oyster *Crassostrea gigas* by importing directly from Japan five t of spats. This effort proved to be successful in time, due to a constant supply of spats from France [2].

*Crassostrea gigas* production increases from one t in 2001 to eight t in 2003, and in 2010 it reached 10 t representing 6.6% of the total shellfish production of Tunisia [3]. Tunisian production of *C. gigas* oyster is distributed exclusively on local markets and occupies the second position among the countries of North Africa, preceded by Morocco (i.e. 10 t for a value of US $35,000 and 284 t for a value of US $538,000 for Tunisia and Morocco in 2010, respectively). This production remains far from France, which is the first producer in Europe with 95,000 t valued at US $428,905,000 for 2010 [3].

The Bizert lagoon, covering an area of 15,000 ha, has been acknowledged for its aquaculture potential; this environment seems to be particularly suitable for oyster farming [4–7]. However, a gradual deterioration of environmental conditions in the Bizert lagoon has been detected since the 1960s, due to increased concentrations of nitrates and phosphates, probably related with population growth and industrial development in the area, which caused eutrophication phenomena [8]. This reality created the necessity of developing experimental oyster farms in maritime areas unaffected by pollution, such as the Port Princes in the Gulf of Tunis [9], to safeguard the products’ quality and promote oyster industry in Tunisia.

Within this context the sexual cycle of *Crassostrea gigas* was compared between farmed populations in continental and marine areas. The obtained data will serve to evaluate the potential success of developing, in near future, oyster farms in marine areas of Tunis.

## Results

### Temperature, salinity and chlorophyll a

**Figure 1:**
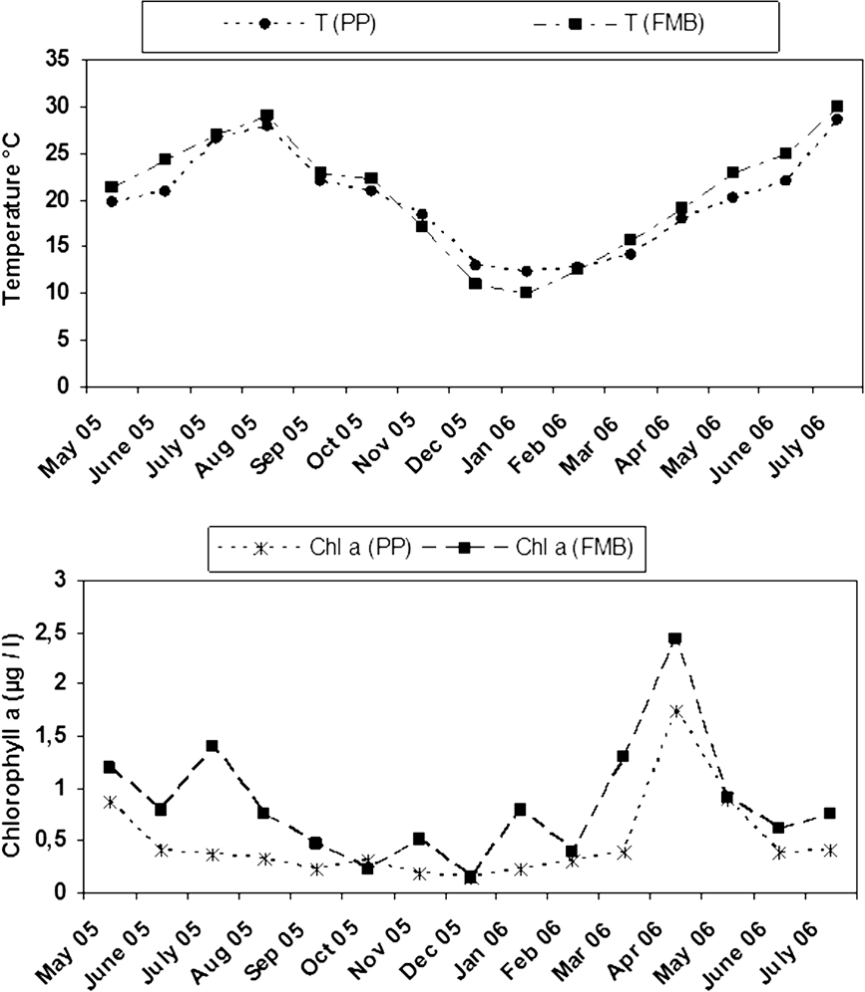
**Monthly variations of temperature (T) and chlorophyll** ***a*** **registered in Port aux Princes (PP) and in Ferme Marine de Bizerte (FMB) in Tunisia.**

Temperature values followed similar temporal trends at both stations, varying from 12.2 to 28.7°C (average at 19.8 ± 5.3°C) at PP (see Methods), and from 10 to 30°C (20.7 ± 6.3°C) at FMB (see Methods) (Figure 1). The average concentration of chlorophyll *a* was lower at PP (0.5 ± 0.4 g l^−1^) than FMB (0.8 ± 0.6 g l^−1^); however similar temporal trends were observed (Figure 1) with minimum values recorded in December (0.15 g l^−1^ at both stations) and maximum ones in April (1.75 g l^−1^and 2.4 g l^−1^, for PP and FMB, respectively).

### Gonadal development stages

**Figure 2:**
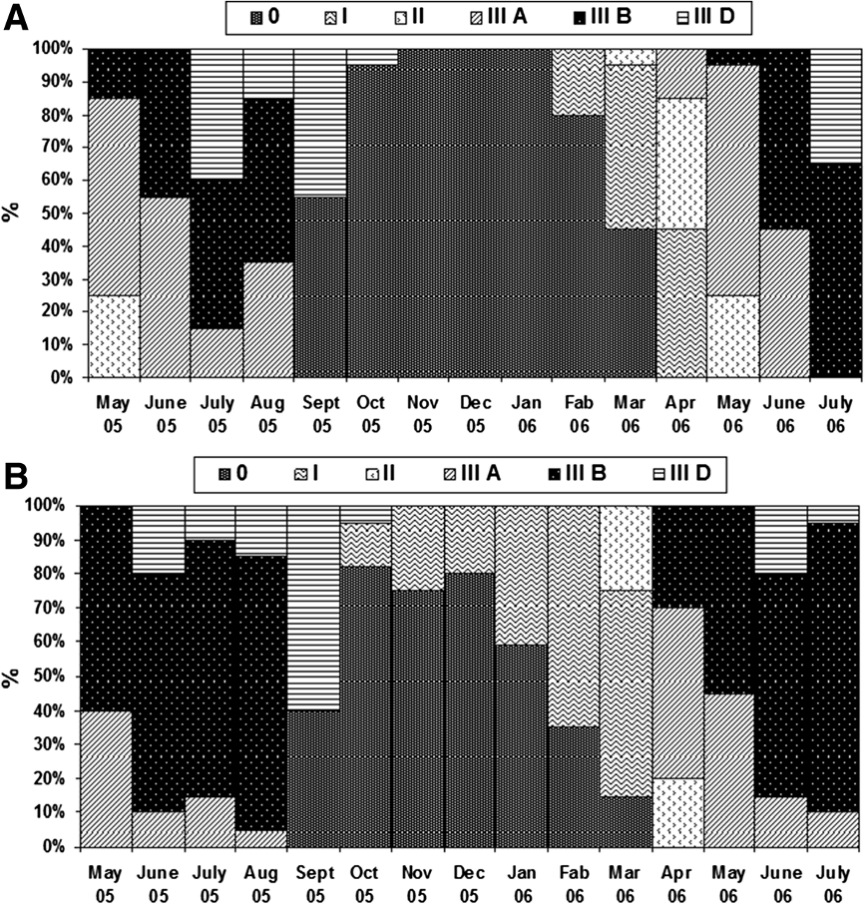
**Variation of gonadic stages (%) of** ***Crassostrea gigas*** **in (A) Port aux Princes (PP) and in (B) Ferme Marine de Bizerte (FMB).** Stages: 0- Undifferentiated gonads, I- Early gametogenesis, II- Gametogenesis, IIIA- Intensive gametogenic activity, IIIB- Gonadal maturation and IIID- Spent gonads.

The six stages of gonadal development previously described for the species were detected at both stations. Their distribution in PP and FMB oyster populations showed rather similar temporal trends (Figure 2A & B), but with some differences related either to the month in which a stage started or ended, or to the percentage contribution of individuals per stage. The following pattern can be generally described for the studied oyster populations. *Crassostrea gigas* was at sexual resting (stage 0, undifferentiated gonads) from September to March at both stations; early gametogenesis (stage I) started earlier in FMB (December) but ended later in PP (April) lasting about three to four months. Gametogenesis (stage II), characterized by the rising number of spermatogonia against the wall of acinus in male and by the accumulation of vitells in the oocyte cytoplasm (previtellogenic oocytes) in females, expands from March to April (FMB) or May (PP). Intense gametogenic activity (stage IIIA) marked by the coexistence of spermatogonia, spermatocytes I and II and spermatides in the follicle of males, and of previtellogenic oocytes, adhering oocyte and peduncular oocytes (mature) in females, was observed from May 2005 to August 2005 and from April 2006 to June 2006, at both PP and FMB. The subsequent stage (stage IIIB) of gonadal maturation (homogenous aspect of gonads after the disappearance of spermatides running toward the center of the follicle in males, presence of numerous peduncular oocytes separated from the follicle lumen or going to be separated in females) was observed from April/May to July/August, whereas spent gonads (ruptured follicles, residual gametes, reappearance of connective tissues between the follicles and haemocytes invading gonadal tubules) were present from June (FMB) or July (PP) to September or October (FMB).

### Sex ratio and hermaphrodism in the studied *C. gigas*populations

**Figure 3:**
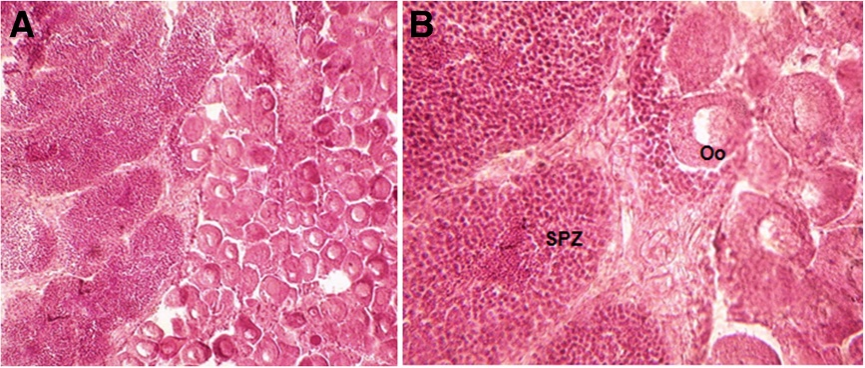
**Simultaneous hermaphroditism in** ***Crassostrea gigas*** **.** (**A**): Hermaphrodism (G = 10 × 10); (**B**): Hermaphrodism (G = 10 × 40) [SPZ = Spermatozoides; Oo = oocyte.

Sex ratio (males/females) ranged from 1.00 to 5.67 in PP and from 1.00 to 4.00 in FMB (Table 1), with an overall mean at 2.23 and 1.6, respectively. According to χ^2^ test results non-significant deviation from 1:1 ratio was detected to the overall population at both sites at 95% confidence level (χ^2^ = 2.96 in PP and χ^2^ = 3.3 in FMB). Three cases of simultaneous hermaphroditism (one at PP and two at FMB), which is rare for *C. gigas*, were detected from the histological examination of the gonads (Figure 3).

**Table 1:** **Sex ratio of the studied** ***C. gigas*** **populations (20 oysters per month and per site)**

Months	PP			FMB		
	Male	Female	Ratio males/females	Male	Female	Ratio males/females
May 2005	12	8	1.5	12	8	1.50
June 2005	14	6	2.33	14	6	2.33
July 2005	15	5	3.00	16	4	4.00
August 2005	17	3	5.67	10	10	1.00
September 2005	10	10	1.00	12	8	1.50
October 2005	-	-	-	10	10	1.00
November 2005	-	-	-	12	8	1.50
December 2005	-	-	-	11	9	1.22
January 2006	-	-	-	6	14	0.43
February 2006	11	9	1.22	8	12	0.67
March 2006	16	4	4.00	7	13	0.54
April 2006	10	10	1.00	16	4	4.00
May 2006	12	8	1.50	15	5	3.00
June 2006	12	8	1.50	9	11	0.82
July 2006	13	7	1.86	7	13	0.54

### Spatio-temporal trends of oocyte diameter

**Figure 4:**
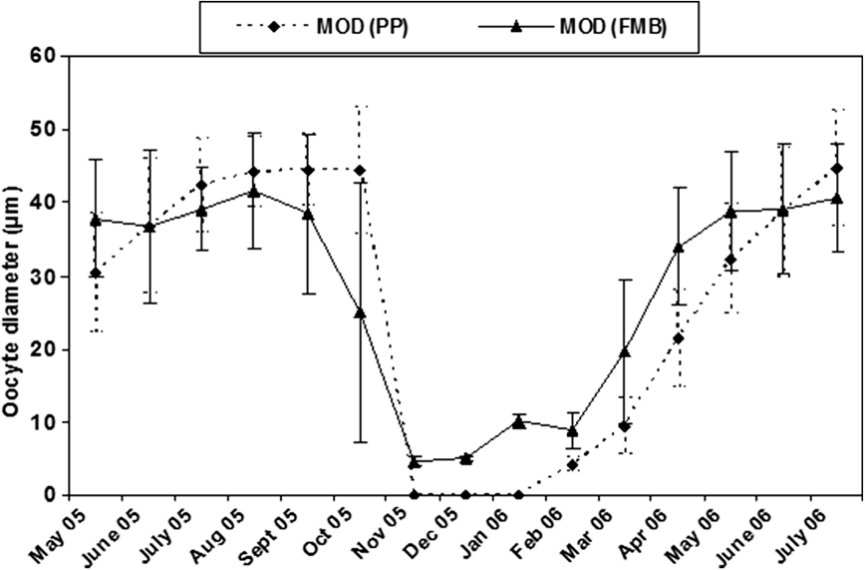
**Average values of oocyte diameters in** ***Crassostrea gigas*** **at Port aux Princes (PP) and Ferme Marine de Bizerte (FMB).**

Mean oocyte diameter (MOD) showed significant temporal variability at both PP and FMB (Figure 4). In PP a gradual increase was recorded from May 2005 to June 2005; thereafter, maximum MOD values were detected until October 2005, where MOD presented a sudden drop as only individuals at the stage 0 were observed from November until January 2006. MOD started again to increase from March 2006 to reach again its maxima in July 2006. A rather similar pattern was detected in FMB; MOD showed increased values until September 2005, and dropped a month earlier in October 2005. In contrast to PP, MOD showed very low, but no-zero values, until January 2006, and then, started to increase to reach its maxima during the period May-July 2006.

Two-way ANOVA detected non-significant differences in MOD between the two studied sites, whereas the relevant temporal differences were significant (Table 2). However, a significant interaction between sites and months was detected to allow further post-hoc comparisons.

**Table 2:** **Spatiotemporal effects on OD, CI and GCI of the studied** ***C. gigas*** **populations**

			OD		CI			GCI		
	Df	Ms	F	***p***	Ms	F	***p***	Ms	F	***p***
**Site**	1	307.94	2.28	0.13	2.28*	1046.14*	0.00*	0.28*	310.9*	0.00*
**Months**	14	70.45	217.8	0.00*	5.60*	6.11*	0.00*	0.42*	7.12*	0.00*
**Site × Months**	29	54.70	144.7	0.00*	1.17*	91.16*	0.00*	0.11*	43.89*	0.00*

* denotes significant differences.

A strong, positive and significant (*p <* 0.05) correlation between MOD and temperature was detected at both stations (r = 0.83 and r = 0.76 for PP and FMB, respectively), whereas the relevant correlation with chlorophyll *a* was weak (r = 0.1 and r = 0.31 for PP and FMB, respectively), though significant (*p* < 0.05).

**Figure 5:**
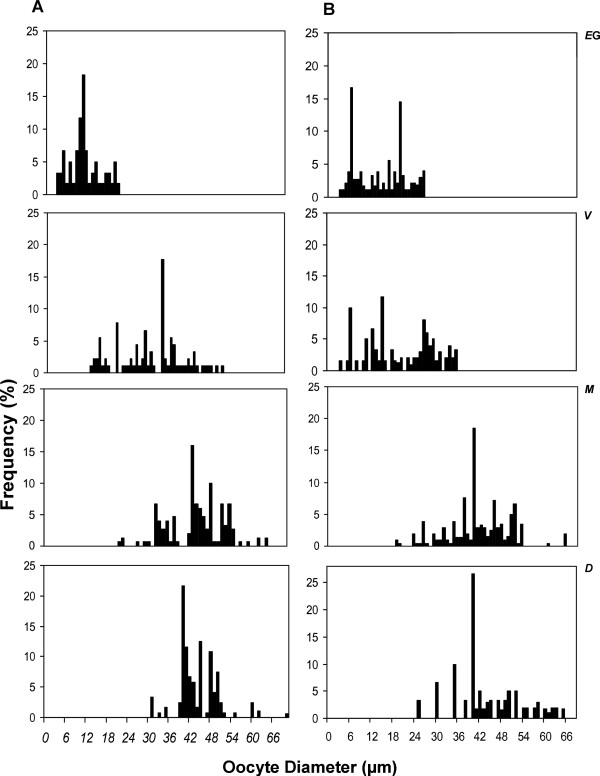
**Oocyte diameter distributions for female** ***Crassostrea gigas*** **in (A) Port aux Prince and in (B) Ferme Marine de Bizerte, representing four sexual maturity stages : Early Gametogenesis (EG), Vitellogenesis (V), Maturing (M) and Degeneration (D).**

Frequency distribution analysis of OD for each maturity stage (Figure 5) showed the gradual increase of oocyte size during sexual maturation. At PP during early gametogenesis most oocytes measured at about 6.5 μm in diameter (February-April); during gametogenesis the bulk of oocytes was 30 μm (March-May), whereas in maturity (May-August and April-July) and degeneration (September) the mode further increased to 40 μm. At FMB, two modes were observed during early gametogenesis (December-March) at 5 and 10 μm in diameter. During gametogenesis (March) oocyte diameter varied from 3.7 to 31 μm with three modes at 6, 10 and 21 μm, respectively; at this month, primary oocytes and previtellogenic oocytes coexisted. During maturation (May-August and April-July) and degeneration (after September), one mode at 40 μm was observed.

### Spatio-temporal trends of the condition and gonadal condition indices

**Figure 6:**
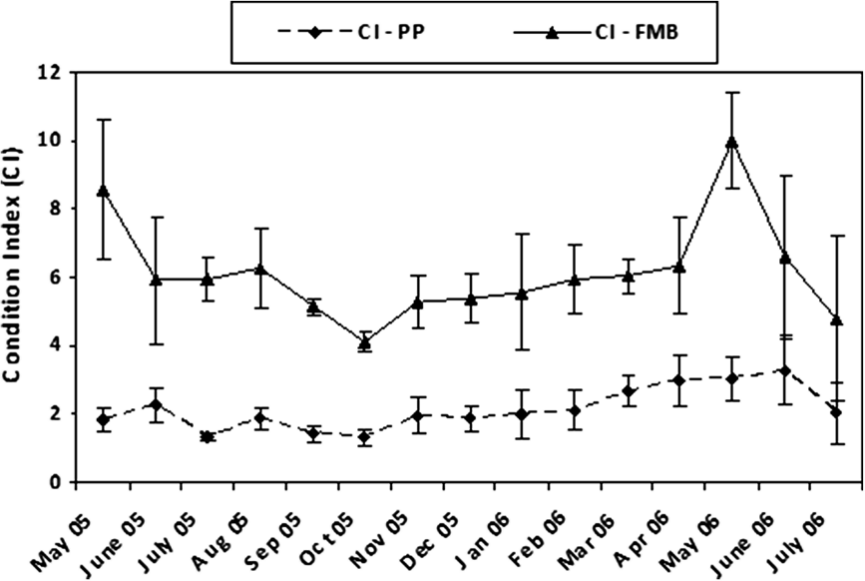
**Variation of condition index (CI) in** ***Crassostrea gigas*** **at Port aux Princes (PP) and a Ferme Marine de Bizerte (FMB).**

**Figure 7:**
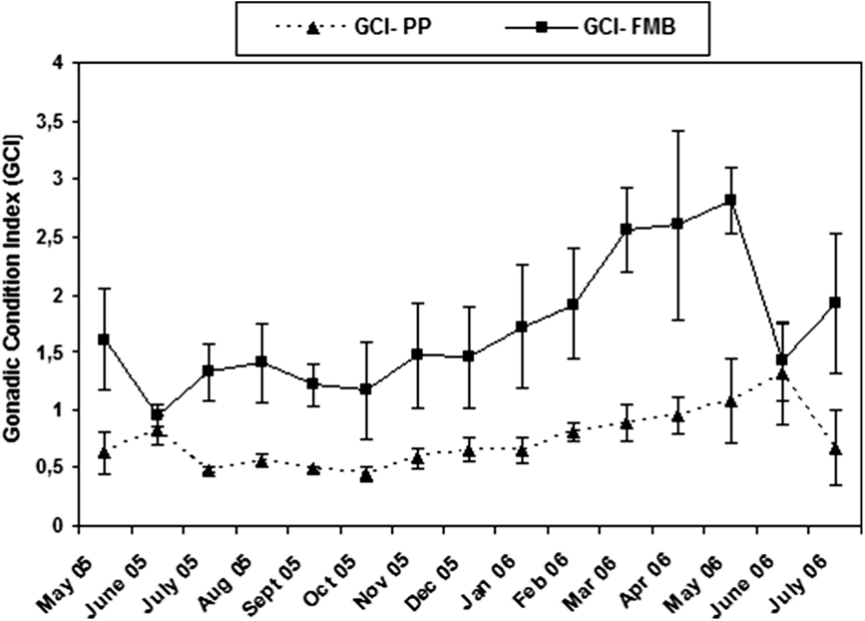
**Variation of gonadosomatic index (GCI) in** ***Crassostrea gigas*** **at Port aux Princes (PP) and a Ferme Marine de Bizerte (FMB).**

CI and GCI showed increased values and diversified temporal pattern in FMB compared to PP sites (Figure 6 & Figure 7). In FMB, maximum CI values were observed in May 2005 and 2006. The index decreased after May 2005 until August, and then, further decreased until October 2005, where it reaches its minima. Thereafter, a gradually increasing trend was observed until April 2006, getting again its maxima in May 2006, and followed by a decrease to its previous values in the next months (Figure 6). In PP the above pattern was modified. In 2005 maximum values were observed in June and minimum ones in July and October; after that month CI followed an increasing trend, reached its maxima in June 2006, and then dropped again in July 2006 (Figure 6).

Two-way ANOVA detected significant differences in CI at both spatial (i.e. between the two studied sites) and temporal scales (Table 2). However, a significant interaction between sites and months was detected to allow further post-hoc comparisons.

A very weak correlation between CI and temperature was detected at PP (r = −0.15, *p* < 0.05), whereas the relevant correlation at FMB was non-significant. CI was also weakly correlated with chlorophyll *a*, at both stations (r = 0.37 and r = 0.24 for PP and FMB, respectively, *p* < 0.05).

GCI index followed the same pattern with CI in both sampling stations, with some slight modifications only in the case of FMB population (Figure 7). Thus, in FMB maximum values were observed from March to May 2006, CI values were much higher in May 2006 compared to May 2005, and the decrease in June 2006 was much more pronounced.

Two-way ANOVA detected significant differences in GCI at both spatial (i.e. between the two studied sites) and temporal scales (Table 2). However, a significant interaction between sites and months was detected to allow further post-hoc comparisons, as the effect of these two factors seems to be overlapping.

At both stations very weak correlations between GCI and temperature (r = −0.15 and r = −0.18 for PP and FMB, respectively, *p* < 0.05), and GCI and chlorophyll *a* (r = 0.30 and r = 0.41 for PP and FMB, respectively, *p* < 0.05) were detected.

## Discussion

The sexual cycle of *C. gigas* is characterized by an extended resting period, lasting from September to March. However, during this period a part of the population reached early gametogenesis at FMB, in contrast to PP where all examined individuals were sexually inactive. Temperature is a widely acknowledged factor influencing oysters’ reproduction; accelerated gametogenesis has been experimentally observed under increasing temperatures [10]. However, as slight differences in temperature were observed between the two sites, with lower values at FMB during winter, other factors may have been also involved. In FMB, increased chlorophyll *a* values were recorded in winter that could have activated the reproductive cycle of oysters, as food availability has been showed to trigger gametogenesis in *C. gigas* from the Atlantic French coast [11] and in *Argopecten irradians* from USA [12]. Early gametogenesis began in February at PP (water temperature: 12.7°C) and in October at FMB (water temperature: 22.2°C), four months in advance. The gametogenic activity of the species initiates at temperatures ranging from 8 to 18°C (see Table 3 summarizing existing information), this range amplifies up to 22°C according to our results.

**Table 3:** **Temperature of gametogenesis initiation and gonads’ maturity for** ***C. gigas*** **populations in different localities**

Localities	Temperature (°C)		References
	Initiation of gametogenesis	Gonads maturity	
Woods Hole (USA)	15-18°C	18°C	[13]
El Grove, Galicia (Spain)	10°C	16°C	[14]
Onagawa Bay, Miyagi (Japan)	8-10°C	16-18°C	[15]
La Tremblade (France)	10-12°C		[11]
Malborough Sounds (New Zealand)	12-13°C	15-17°C	[16]
Marennes Oléron Bay (France)	8-12°C	16-18°C	[17]
Bizert lagoon (Tunisia)	14-15°C	16-20°C	[18]
Sonora (Mexico)	14°C		[19]
**PP, Gulf of Tunis (Tunisia)**	**12.7°C**	**20.2°C**	**Present work**
**FMB, Bizert lagoon (Tunisia)**	**22.2°C**	**19.2°C**	**Present work**

Vitellogenesis started in March at both sites; however it was shorter and more intense in FMB, where increased temperature and chlorophyll *a* values were recorded at that period. This conforms to previous data suggesting that the reproductive strategy of *C. gigas* is strongly affected by water temperature regulating the speed and thus, the length of gametogenesis, and also by nutrient content affecting the intensity of different gonadal stages [11]. Sexual maturity in *C. gigas* started in spring, when temperature and/or chlorophyll *a* values further increased, being more intense and in advance in FMB. In this station, water temperature was higher during this period coinciding with the oyster’s sexual maturity, as well as chlorophyll *a* values, indicating the nutrient-rich environment of Bizert lagoon, compared to the open sea. It seems therefore that the warm water and the higher food availability of the Bizert lagoon favors the gametogenic cycle of *C. gigas* which is earlier and more intense than the gulf of Tunis (PP). Maturity reached at temperatures from 19-20°C, which is well within the known temperature range for the species (see Table 1 summarizing existing information).

Comparing the gametogenic cycle of *C. gigas* with previous data from the Bizert lagoon [18] some differences came up. The reproductive pattern observed in 2002–2003 was rather similar with that currently observed at PP (discontinuous gametogenesis, initiation in February), but not with that from the same location (in 2005–2006, gametogenesis initiated in October and was continuous at FMB). Such spatiotemporal differences in the reproductive pattern of the species may be explained by relevant environmental differences. Indeed, the concentration of chlorophyll *a* was almost twice as high in summer 2005 compared with 2002, probably inducing the recorded continuity of the gametogenic cycle. The duration of gametogenic cycle in *C. gigas* seems to follow a latitudinal gradient, controlled mainly by temperature [10], which may have a positive effect, either directly by affecting the metabolic rate of the species, or indirectly by enhancing food availability, as it has been showed for other bivalve species [20].

Temperature and food availability are among the most determining factor of gametogenesis in bivalves, which seems to be controlled by the spring phytoplankton blooms, and also, by phytoplankton concentration in winter, which determines the reserve storage [21–24]. Oysters under poor diet conditions, spent entirely their gametes and presents a short period of absorption, whereas under rich ones, they spent partially and follow a prolonged restoration period [11]. The absorption of gonads constitutes a “self-cleaning” process during which massive phagolysis of gametes takes place [11]; its output can be used either to cover basal metabolism [25], or to generate glycogen reserves which will be used to the next gametogenic cycle. The above may explain the observed differences considering the duration of the gonads absorption period between the studied oyster populations.

Both *C. gigas* populations studied showed an, overall, equal distribution of sexes, despite the predominance of males in several months, though these results must be cautionary interpreted as only 20 oysters per month and site have been examined. For the same species populations in Japan, sex ratio has been showed to be affected by the oyster length and age [26], with a predominance of males in both small, early stage and large, older oysters. These data have been interpreted as evidences of rhythmical hermaphroditism [26]. However, further research is required to assess relevant patterns in Mediterranean populations of *C. gigas*.

Oocyte diameter of *C. gigas* at sexual maturity varies around 34.9 ± 9.8 μm with a maximum of 61.4 μm [27] or even 70 μm [28], for French populations of the species. From the Sea of Japan even larger oocytes have been reported, reaching 80 μm [29]; these voluminous oocytes have been attributed to the environmental conditions and the genetic structure of the studied population. In the present study average size of oocytes in maturity was larger in PP (44.6 ± 7.9 μm) than FMB (40.7 ± 7.4 μm) and it was strongly correlated with temperature. This result is in contradiction with previous data from other bivalve species reporting a positive relationship between food availability and oocyte diameter [30]. However, the ability of *C. gigas* populations to produce small oocytes when growing under plenty of food has been also demonstrated [31].

Oysters CI is directly related with the production of gametes, the increase of the somatic tissue, the shell growth and the secretion of mucus [32]. CI increased during the maturation phase and decreased when gametes are released and in the beginning of the sexual repose phase. During this latter phase, the species accumulate the necessary metabolites to start the next sexual cycle [33], and this gradual storage of organic matter may explain the subsequent increment of CI.

GCI results generally coincide with CI. GCI increased at the maturation phase and decreased when gonads are spent and during the sexual repose phase; these results are in accordance with the histological examination of *C. gigas* gonads. GCI temporal trends suggest the precocity and the faster gametogenic cycle of *C. gigas* in the Bizert lagoon compared with the Gulf of Tunis. However, as both indices presented weak correlation with temperature and chlorophyll *a* other factors must be examined to explain the observed differences between the studied sites.

## Conclusions

The results obtained allowed the comparison of the sexual cycle between oysters farmed in marine and lagoon environments. The oyster’s gametogenic activity was successfully completed in both culture sites, following a clear seasonal pattern. Histological analysis, OD, CI and GCI data showed that the gametogenic cycle of *C. gigas* preceded in the lagoon environment, whereas the reproductive potential of the species was also increased in the latter habitat. These differences are probably related with relevant differences in thermal regime and trophic status of the two contrasting environments. Overall, the obtained results provide further evidence of the invasive character of *C. gigas* as the species is able to adapt in different environments. The development of oyster farms in marine localities of Tunis seems promising although more favorable conditions for *C. gigas* farming (i.e. increased chlorophyll *a* concentrations) occurs in the Bizert lagoon, as suggested by CI and GCI results, which were by far higher in the oysters breeding in the lagoon.

## Methods

### Study area and field sampling

**Figure 8:**
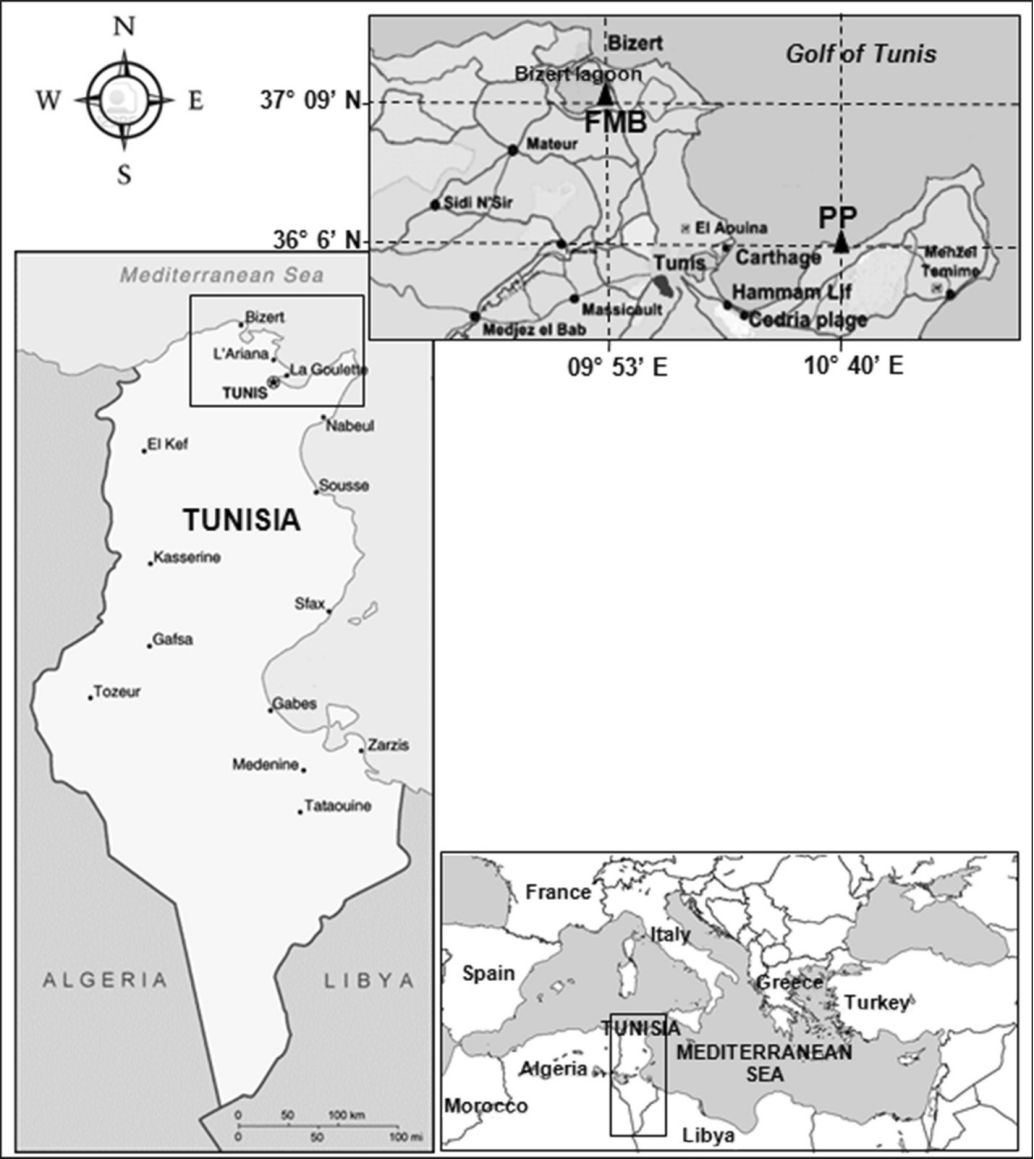
**Sampling of** ***C. gigas*** **sites localisation in the Bizert lagoon (FMB) and the gulf of Tunis (PP).**

One oyster farming ground and an oyster purification site were selected as sampling sites: “Ferme Marine de Bizerte” (FMB), located in the southeast of the Bizert lagoon and “Port aux Princes” (PP), located in the eastern Gulf of Tunis, respectively (Figure 8). In both sites, oysters were placed in pockets suspended from rafts at 2 m depth.

Field samplings were completed from May 2005 to July 2006 on monthly basis at both sites and included the collection of *C. gigas* specimens (50 adult specimens per month and per site) measuring 8–10 cm in length (anterior-posterior distance measure) to assess the sexual cycle of the species, and the estimation of main physical and chemical parameters of the water column. Temperature was measured *in situ* at 1 m depth using an electronic thermometer (VWR, Vienna, Austria). Chlorophyll *a* was estimated applying the fluorometric method by measuring the fluorescence before and after acidification of the methanolic pigment extract [34].

### Histological analysis and oocyte diameter

In the laboratory, 20 of the collected *C. gigas* specimens, per month and per site, were dissected. Each visceral mass was fixed in the Bouin’s solution and thereafter dehydrated in a series of increasing concentrations of ethanol. Dehydrated samples were cleared and embedded in paraffin following a standardized procedure [27]. Sections (6 μm thick) were mounted on glass slides and stained with Groat’s hematoxylin and eosin solution [35]. Each section was examined under light microscopy to determine sex and gonadal stage using the six-stages scale of gonadal development previously described for the species [13, 36].

Each month, five females from each station were randomly selected to determine Oocyte Diameter (OD). Approximately the diameter of 100 oocytes, with a visible nucleolus, were measured by female [15] using a microscope equipped with an ocular micrometer.

According to OD and histological characteristics of the gonad the sexual maturity of females was classified to the four-stages of development, i.e. Early Gametogenesis (EG), Vitellogenesis (V), Maturing (M) and Degeneration (D) previously described for *C. gigas* [27]. The size of degenerating oocytes, present after spawning, was not measured because they were torn or broken.

### Condition and gonadal condition indices

Thirty of the collected *C. gigas* specimens, per month and per site, were used to calculate the Condition Index (CI) of the species, estimated as the percent ratio of flesh to shell dry weight [37]. The same specimens were used to estimate the Gonadal Condition Index (GCI), which was defined as the percent ratio of the visceral mass (gonad mixed with the hepatopancreas) to shell dry weight [38]. To assess dry weights, tissues and shells were dehydrated by maintained in oven at 60°C for 72 hrs [22]; then, weights were measured using an electronic scale (precision 0.001 g).

### Statistical analysis

Two-way analysis of variance was used to test for temporal (among sampling months) and spatial (between sampling sites) effects on mean CI, GCI and OD values. Least Significant Differences test (LSD) was used for post hoc comparisons at 5% significance level. Correlations between the measured abiotic variables (temperature and chlorophyll *a*) with OD, CI and GCI were also calculated. A χ^2^ test was used to assess whether oyster individuals were equally distributed among sexes. All statistical tests were performed using STATISTICA 10.0 (StatSoft, Tulsa, USA) software.

## Abbreviations

PP: Port aux princes
FMB: Ferme marine de Bizerte
GCI: Gonadal condition index
CI: Condition index
OD: Oocyte diameter
MOD: Mean oocyte diameter
LSD: Least significant differences test.
